# The Cks1/Cks2 axis fine-tunes Mll1 expression and is crucial for MLL-rearranged leukaemia cell viability

**DOI:** 10.1016/j.bbamcr.2017.09.009

**Published:** 2018-01

**Authors:** William Grey, Adam Ivey, Thomas A. Milne, Torsten Haferlach, David Grimwade, Frank Uhlmann, Edwige Voisset, Veronica Yu

**Affiliations:** a Department of Medical and Molecular Genetics, King's College London, Faculty of Life Sciences and Medicine, London, UK; b MRC Molecular Haematology Unit, Weatherall Institute of Molecular Medicine, NIHR Oxford Biomedical Research Centre Programme, University of Oxford, UK; c MLL Munich Leukemia Laboratory, Munich, Germany; d Chromosome Segregation Laboratory, The Francis Crick Institute, London, UK

**Keywords:** *CKS1B*, *CKS2*, MLL1, Wnt, MLL-fusion proteins, SKP2/CKS1 inhibitor

## Abstract

The Cdc28 protein kinase subunits, Cks1 and Cks2, play dual roles in Cdk-substrate specificity and Cdk-independent protein degradation, in concert with the E3 ubiquitin ligase complexes SCF^Skp2^ and APC^Cdc20^. Notable targets controlled by Cks include p27 and Cyclin A. Here, we demonstrate that Cks1 and Cks2 proteins interact with both the Mll^N^ and Mll^C^ subunits of Mll1 (Mixed-lineage leukaemia 1), and together, the Cks proteins define Mll1 levels throughout the cell cycle. Overexpression of *CKS1B* and *CKS2* is observed in multiple human cancers, including various *MLL*-rearranged (*MLLr*) AML subtypes. To explore the importance of MLL-Fusion Protein regulation by CKS1/2, we used small molecule inhibitors (MLN4924 and C1) to modulate their protein degradation functions. These inhibitors specifically reduced the proliferation of *MLLr* cell lines compared to primary controls. Altogether, this study uncovers a novel regulatory pathway for MLL1, which may open a new therapeutic approach to *MLLr* leukaemia.

## Highlights

• Mll1 is controlled by the Cks1/Cks2 axis.
• Opposing effects: Cks1 promotes Mll1 degradation, while Cks2 stabilises Mll1.
• Cks1/Cks2 impact on Wnt signalling through Mll1.
• *CKS1* and *CKS2* are overexpressed in *MLL*-rearranged AML.
• Pharmacological inhibition of SCF^SKP2-CKS1^ is cytotoxic for *MLL-*rearranged AML.

## 1 Introduction

The Cks proteins are small phospho-adapters required for correct CDK substrate recognition (but not kinase activity), and more precisely, for the specification of multisite phosphorylation [1], [2]. Further Cks functions in transcription and protein degradation have been reported in yeast [3], [4] and mammals [5], [6], [7]. Indeed, it has been shown that Cks1 and Cks2 associate with two ubiquitin E3 ligase complexes, SCF^Skp2^ and APC^Cdc20^, thereby regulating degradation of Cyclin A and the CDK inhibitor p27 [7], [8]. Despite redundant functions between Cks1 and Cks2 [5], [9], [10], [11], sequence divergence in the Skp2-binding region results in opposing roles during degradation of the Skp2 target p27. Indeed, Cks1 brings p27 to Skp2, facilitating p27 ubiquitination and degradation, while Cks2 protects p27 from Skp2 interactions and stabilises p27 [7], [8], [12]. Whereas both *Cks1*^*−/−*^ and *Cks2*^*−/−*^ mice are viable [7], [13], the *Cks1* and *Cks2* double knockout is embryonic lethal after the morula stage, indicating that Cks1 and Cks2 are essential for embryonic development [5]. Although many *Cks1*^*−/−*^ and *Cks2*^*−/−*^ mouse model phenotypes are due to altered p27 regulation, loss of *Cks1* in *Eμ-Myc* transgenic mice (a model of human Burkitt's lymphoma) reduces cancer progression independent of p27 regulation [14], demonstrating a role for Cks1 beyond p27 regulation. In further studies of their tumorigenic potential, *Cks1* and *Cks2* have not only been described to be under the control of c-Myc, but also of B-RAF and Cyclin D1 oncoproteins [15]. Moreover, *CKS1B* and *CKS2* are frequently overexpressed in various cancers [16], [17], [18], [19], including multiple myeloma [20], [21] and breast cancer [6], [22], [23], correlating with increased proliferation and poor prognosis.

MLL1 is a histone methyltransferase, which modulates a gene expression signature important for embryonic and haematopoietic stem cell development [24], [25], [26]. The MLL1 protein is cleaved by Taspase 1 into N-terminal (MLL^N^) and C-terminal (MLL^C^) fragments [27], [28], which require phosphorylation and inter-molecular interaction for full activity [27], [29]. Furthermore, bimodal degradation of MLL1 by the SCF^SKP2^ and APC^CDC20^ complexes results in a cell cycle-dependent, biphasic expression profile [30]. The human *MLL1* gene is chiefly known for its involvement in chromosomal translocations driving mixed-lineage leukaemias [31]. Leukaemic rearrangements fuse the N-terminal portion of MLL1 with a variety of translocation partners to produce a mature MLL-Fusion Protein (MLL-FP), which consequently omits the MLL1 C-terminal domains [32], [33]. Common *MLL1* translocation partners include *AFF1/AF4*, *MLLT4/AF6*, *MLLT3/AF9*, *MLLT10/AF10*, *ELL*, *and MLLT1/ENL* [34]. Mechanistically, MLL-FPs show diminished interactions with the SCF^SKP2^ and APC^CDC20^ complexes, resulting in their stabilisation [30], and the building of diverse transcriptional complexes, which override the normal histone methyltransferase activity of MLL1 [28]. These alterations lead to stable expression of developmentally important target genes (e.g. *Hox* genes), and aberrant activation of various signalling pathways [35]. Additionally, stabilisation of wild-type (WT) MLL1 protein in *MLLr* cell lines has been revealed as an important route for competing with, and suppressing the oncogenicity of, MLL-FPs [36]. *MLL*-rearranged (*MLLr*) leukaemias are currently treated by chemotherapy, but with 5 year survival rates below 50% and 20% in paediatric and adult cases respectively, there is a critical need for more effective therapies [37].

In this study, we identified Mll1 as a Cks1/Cks2 interactor and show that Mll1 stability is controlled by the Cks proteins. Collectively, our data demonstrates a role for Cks proteins in the regulation of Wnt signalling through Mll1. This previously unknown role for the CKS1/CKS2 axis has consequences for normal MLL1 function but, unexpectedly, also for MLL-FP leukaemic activity. These findings offer a new potential therapeutic target for the treatment of poor prognosis acute leukaemias.

## 2 Materials and methods

### 2.1 Cell culture and patient samples

Mouse Embryonic Fibroblasts (MEFs) were isolated from day E13.5 embryos, and cultured in DMEM supplemented with 10% Foetal Bovine Serum (FBS) and 5% penicillin/streptomycin (ThermoScientific, Loughborough, UK) as previously described [8], and were spontaneously immortalised by the 3T3 protocol. In accordance with previously reported data [8], we confirmed that *Cks1*^*−/−*^ MEFs have a slower cell cycle, increased G1 phase population and p27 protein level when compared to wild-type (WT) control. Conversely, *Cks2*^*−/−*^ MEFs cycle faster, with an increased S phase population, lower p27 protein level and increased γH2AX level when compared to WT control (Fig. S1).

ML-2 (DSMZ, Braunschweig, Germany; ACC15), THP-1 (DSMZ; ACC16), KOPN-8 (DSMZ; ACC552), ML-1, and RS4; 11 cell lines and peripheral blood mononuclear cells (PBMCs) [38], [39] were cultured in RPMI 1640 (ThermoScientific) with 10% FBS and 5% penicillin/streptomycin.

Diagnostic peripheral blood or bone marrow cDNA samples were obtained from the MLL Munich Leukemia Laboratory. PBMCs and cord blood mononuclear cells were obtained from healthy donors and separated using Ficoll-Paque Plus as per the manufacturer's instructions (GE Healthcare, Amersham, UK). CD34^+^ cells were isolated using the EasySep Human CD34 positive selection kit (Stem Cell Technologies, Cambridge, UK), and cultured in StemSpan SFEM II medium for expansion supplemented with hSCF (300 ng/ml), hTPO (20 ng/ml) and hFLT3L (300 ng/ml) for optimal proliferation (Peprotech, London, UK). RT-qPCRs were performed according to standard Europe Against Cancer conditions [40].

### 2.2 Cell cycle synchronisation

MEFs were synchronised in G1 phase by serum starvation (1% FBS), in S phase by double thymidine block (2 mM thymidine; Sigma-Aldrich, Dorset, UK), and in M phase by nocodazole block (40 ng/μl nocodazole; Merck Millipore, Watford, UK) for 12 h.

### 2.3 Cell transfection

All cells were transfected by nucleofection using the Amaxa nucleofector system (Lonza, Slough, UK) with either plasmid DNA (0.5-2 μg) or siRNA (0.5-1 μM). MEFs were transfected using the P4 Primary Cell Kit and program CZ-167, and *MLL*-translocation cell lines using the Cell Line kit L and program A-020. The FLAG-MLL1 plasmid was a kind gift from Prof. Thomas Milne. All RNA interference knockdowns were performed with two independent siRNAs, and siRNA sequences are as follows: Non-Targeting Control (Mouse) 5′-UGGUUUACAUGUCGACUAA-3′, 5′-UGGUUUACAUGUUGUGUGA-3′, 5′-UGGUUUACAUGUUUUCCUA-3′, 5′-UGGUUUACAUGUUUUCUGA-3′ Dharmacon (D-001810-10-20); Mll1 (11) 5′-GCACAGUGGUCUCACGAUU-3′ Dharmacon (J-040631-10); Mll1 (14) 5′-CUGUUGAAUUCUCGGACUA-3′ Dharmacon (J-040631-11); Non-Targeting Control (Human) 5′-UGGUUUACAUGUCGACUAA-3′, 5′-UGGUUUACAUGUUGUGUGA-3′, 5′-UGGUUUACAUGUUUUCCUA-3′, 5′-UGGUUUACAUGUUUUCUGA-3′ Dharmacon (D001810-10-20); CKS1B (A) 5′-CGACGAGGAGUUUGAGUAUUU-3′ [6]; CKS1B (B) 5′-ACCAGAACCUCACAUCUUGUU-3′ [6]; CKS2 (A) 5′-CUGCAAGUAGGUUACUGUA-3′ [18]; CKS2 (B) 5′-GUUUGUAUGUUGCAUUUAATT-3′ [11].

### 2.4 Immunofluorescent staining, ImageStream^X^ and confocal microscopy

For ImageStream^X^ analysis, cells were fixed in 4% paraformaldehyde, permeabilised in 0.5% Triton X-100, and incubated overnight with a pan-β-catenin antibody (Cell Signalling Technology, Hitchin, UK (CST; #8480)) followed by anti-Rabbit-FITC (ThermoScientific). Cells were resuspended in PBS containing 2 mM EDTA and 5 μg/ml DAPI. Stained cells were analysed on an ImageStream^X^ Mark II imaging flow cytometer using the INSPIRE application (Amnis, Seattle, WA, USA). Analysis was carried out using the IDEAS software (Amnis). Cells were selected as follows: Single cells, Focused cells, Double positive (β-catenin/DAPI). Final selected cells were analysed using the feature and mask: Similarity_Object(M01,Brightfield,Tight)_β-catenin_DAPI, which calculates Similarity (logarithmic transformation of Pearson's correlation coefficient) for β-catenin and DAPI co-localisation within the cell [41] (Fig. S3). A minimum of 10,000 cells was present in the final gate (double positive). Fisher's discriminant ratio (Rd Median) was calculated to measure nuclear translocation of β-catenin as follows: Rd = (Median sample − Median negative control)/(Median absolute deviation sample + Median absolute deviation negative control). Confocal microscopy for γH2AX was carried out as previously described [8].

### 2.5 Real-time quantitative PCR

Total cellular RNA was extracted from cells using the RNeasy Mini Kit (Qiagen, Crawley, UK), with on-column DNase I digestion, as per the manufacturer's instructions. Quantitative PCR analyses were carried out using either the Quantitect SYBR Green RT-PCR kit (MEFs – Qiagen) or the TaqMan Universal PCR Master Mix kit (*MLL*-translocation cell lines – ThermoScientific). All SYBR Green and TaqMan assays were run on a 7900HT Real-Time PCR system (Applied Biosystems, UK). Primers are listed in Table S1.

### 2.6 TCF/LEF reporter assays

Both MEFs and *MLL*-translocation cell lines were co-transfected with 5 μg M50 Super 8x TOPFlash (#12456; Addgene) or control M51 Super 8x FOPFlash (#12457; Addgene) and 100 ng Renilla control (pRL-TK; Promega, Southampton, UK). Luciferase activity was measured using the Dual Luciferase Assay System (Promega). Relative Luciferase Activity (RLA) was calculated as Luciferase/Renilla signal.

### 2.7 Cell viability and apoptosis assays

Cells were seeded in triplicate in 96-well plates (1 × 10^5^ cells/ml) with or without different concentrations of inhibitor (SKP2 E3 Ligase Inhibitor C1 or NAE inhibitor MLN4924; Merck Millipore). Cell viability was assessed using AlamarBlue (ThermoScientific), according to the manufacturer's instructions, 48 h post-treatment.

For the evaluation of apoptosis, cells were stained with Annexin V-FITC and PI (Biolegend, London, UK) following manufacturer's instructions. A minimum of 20,000 cells were assayed for each condition on a flow cytometer (BD Canto), and analysed using FlowJo software (Tree Star, Switzerland).

### 2.8 Cell cycle analysis

Cells were pulsed with 10 μM EdU for 60 min, and fixed in 4% paraformaldehyde. EdU incorporation was assessed with the Click-iT EdU AlexaFluor 647 kit (ThermoScientific), following manufacturer's instructions. At least 20,000 cells were acquired using a BD Canto flow cytometer and analysed with FlowJo software.

### 2.9 Immunoblot analysis

Nuclear and cytoplasmic extracts were prepared using the ThermoScientific NER kit as per manufacturer's instructions. Whole cell extracts were prepared using RIPA buffer. Western blots of MLL^N^ and MLL^C^ subunits were carried out on NuPAGE 3–8% Tris-Acetate gels (ThermoScientific) as per the manufacturer's instructions.

Primary antibodies used in this study were as follows: H3 (06-755) and γH2AX (JBW301) (Merck Millipore); Actin (#3700), pan-β-catenin (#8480), Tubulin (#2128), Pin1 (#3722), phospho-AKT Ser473 (#4060) and AKT (#4691) (CST); p27 (610241) (BD Biosciences, Oxford, UK); MLL1 (A300-086A), AF9 (A300-596A), AF6 (A302-199A) and ENL (A302-268A) (Bethyl, UK); MLL^C^ (sc-374,392), pan-Cks (sc-6238), CDK2 (sc-163), Cleaved-Caspase 3 (sc-7272) and normal IgG (Santa Cruz, CA, USA); and Cks1 (37-0200) and Cks2 (37-0300) (ThermoScientific).

### 2.10 Co-immunoprecipitation assay

Whole cell lysates were pre-cleared with protein G sepharose beads (GE Healthcare), and incubated overnight at 4 °C under constant rotation, with either anti-FLAG M2 affinity gel (A2220; Sigma-Aldrich) or protein G sepharose beads with the relevant antibody. 2% of each pre-cleared sample was kept as input control. Beads were washed in RIPA buffer, and protein complexes were eluted with 3X FLAG peptide (F4799; Sigma-Aldrich) or in Laemmli buffer.

### 2.11 Colony formation assay

Indicated cell numbers were plated in 2 ml cytokine supplemented methylcellulose (Methocult H4434, Stem Cell Technologies) and incubated for 14 days before scoring for presence of phenotypic colonies according to the manufacturer's instructions.

## 3 Results

### 3.1 Cks1 and Cks2 control Mll1 protein levels and influence Mll1-dependent gene expression

**Fig. 1:**
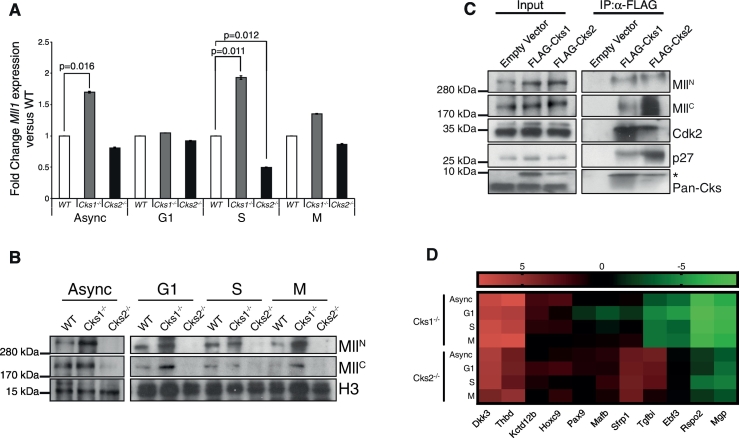
The cell cycle dependent regulation of Mll1 is altered in *Cks*-deficient MEFs, with downstream consequences on Mll1-dependent gene expression. (A) RNA levels of *Mll1* in asynchronous (Async) and synchronised *Cks*-deficient MEFs. All cell cycle phases are represented as fold change versus WT control. A Student's *t*-test was used to assess significance of differences (N = 3). (B) Western blots for nuclear extracts of asynchronous and synchronised MEFs. Histone H3 was used as a nuclear loading control. (C) Whole cell lysates from WT MEFs overexpressing FLAG-tagged Cks1 or Cks2 were immunoprecipitated using FLAG-agarose slurry. Western blots for p27 and Cdk2 were used as positive controls for co-immunoprecipitation. * indicates FLAG-tagged Cks. Western blots are representative of 3 independent experiments. (D) Heat map of Mll1 target gene expression in *Cks*-deficient MEFs. RNA extracted from asynchronous (Async) and synchronised MEFs was analysed for the top downregulated Mll1 target genes as designated by Wang et al. [26]. Two independent control genes (*Actin* and *Gapdh*) were used to measure relative RNA abundance. *Actin* controlled values are represented as Log2 fold change expression versus WT control from the same cell cycle stage. Data analysis was performed using GraphPad Prism 7 software.

The first evidence that the Mll1 protein may be under the control of the Cks1/Cks2 axis is based on Mll1 biphasic, cell-cycle dependent regulation by the SCF^Skp2^ and APC^Cdc20^ ubiquitin E3 ligase complexes, both of which require Cks for degradation of a portion of their known targets. Further to this, in silico analysis revealed that Mll1 contains multiple putative Cks1 minimum consensus sites ([FILPVWY]xTP) on both N- and C-terminal subunits [2], [42]. To investigate whether Mll1 protein levels, similar to p27 [8], are controlled by the Cks1/Cks2 axis, we used WT, *Cks1*^*−/−*^ and *Cks2*^*−/−*^ MEFs (Fig. S1), that were synchronised in G1, S and M phases of the cell cycle, to analyse *Mll1* expression at both the RNA and protein levels (Fig. 1A-B). *Mll1* transcript abundance was significantly altered in S phase for both *Cks1*^*−/−*^ (> 2-fold higher; p = 0.011) and *Cks2*^*−/−*^ (≥ 2-fold lower; p = 0.012) compared to WT. The same trend was observed in asynchronous cells, however, only the increased mRNA levels in *Cks1*^*−/−*^ MEFs was significantly different from control levels (≥ 1.5-fold higher; p = 0.016; Fig. 1A).

Mll1 protein levels do not directly correlate with mRNA levels, and the predominant route of Mll1 regulation has been reported to be via protein degradation [30]. To examine whether Cks proteins modulate Mll1 protein stability, we used western blotting to compare the levels of both Mll^N^ and Mll^C^ subunits in *Cks1*^*−/−*^ and *Cks2*^*−/−*^ MEFs. This revealed increased Mll^N^ as well as Mll^C^ levels in *Cks1*^*−/−*^ MEFs, both in asynchronous cells and at synchronised cell cycle stages. The relative increase was most striking in G1 and M phase cells and less pronounced in S phase cells. In contrast, both Mll1 subunits were almost undetectable in *Cks2*^*−/−*^ cells when compared to WT controls (Fig. 1B). Remarkably, this is reminiscent of the cell cycle regulator p27, whose degradation by SCF^Skp2^ is promoted by Cks1 and is protected from degradation by Cks2 [8].

To determine whether Cks proteins directly interact with Mll1 to influence its stability, or this effect was an indirect downstream consequence of Cdk activity changes (e.g. as seen for γH2AX [8]), we performed co-immunoprecipitation assays. WT MEFs overexpressing FLAG-tagged Cks1 or Cks2 were lysed and incubated with FLAG-conjugated agarose beads, to pull down Cks proteins. In these conditions, Cks1 and Cks2 were consistently found to pull down both Mll^N^ and Mll^C^ subunits (Fig. 1C). Altogether, these results strongly suggest that Cks proteins regulate Mll1 protein levels in a fashion analogous to their opposing roles in p27 degradation. Indeed, Cks1 would promote Mll1 degradation, while Cks2 would act to stabilise Mll1 by protecting it from Cks1-mediated degradation.

The histone methyltransferase function of Mll1 is critical for promoting transcription of an array of target genes, with *Mll1*^*−/−*^ MEFs showing significant reductions in expression of these target genes compared to WT controls [26]. Comparison of WT versus *Cks1*^*−/−*^ or *Cks2*^*−/−*^ total RNA, from asynchronous and synchronised cells, revealed major alterations in those Mll1 target genes defined by Wang et al. [26] as most frequently downregulated (e.g. *Dkk3*, *Thbd, Hoxc9* and *Mgp*; Fig. 1D). Interestingly, the differences in Mll1 target genes underlined the overlapping and independent functions of Cks1 and Cks2. Whereas the changes in *Dkk3*, *Thbd*, *Rspo* and *Mgp* were similar between *Cks1*^*−/−*^ and *Cks2*^*−/−*^ cells, a subset of Mll1 target genes (e.g. *Sfrp1*, *Tgfbi* and *Ebf3*) was differentially regulated between *Cks* knockout cells. This indicates that the alteration of Mll1 protein levels in *Cks*-deficient MEFs has a direct impact on the primary cellular function of Mll1.

### 3.2 Opposing roles of Cks1 and Cks2 in Wnt signalling through Mll1 regulation

**Fig. 2:**
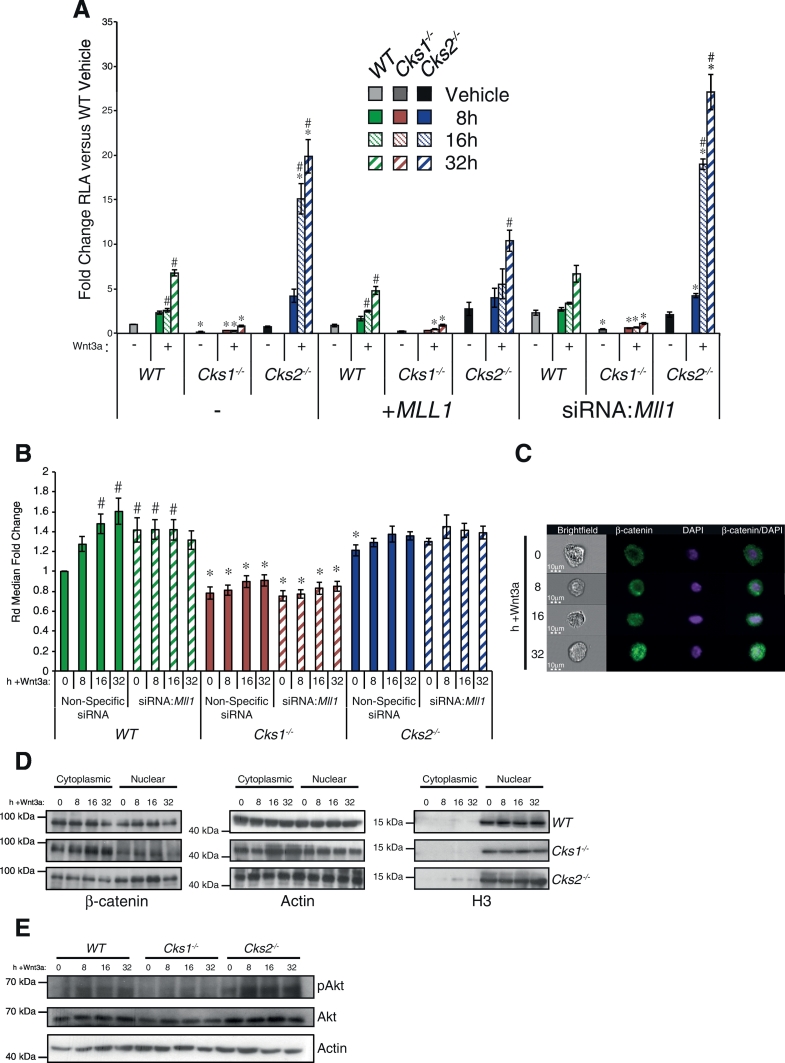
Wnt signalling alterations in *Cks*-deficient MEFs are partially rescued by *Mll1* alterations. (A) Untreated, *MLL1* overexpressing, and *Mll1* knockdown MEFs were treated with 50 ng/ml Wnt3a and assayed for relative luciferase activity (RLA) for the indicated time points. Values are represented as fold change RLA versus endogenous WT control. A Student's *t*-test was used to determine significance of differences (N = 3). * indicates significant differences versus WT control (p < 0.05). # indicates significant differences versus genotype specific control (p < 0.05). (B) WT and *Cks*-deficient MEFs were transfected with either non-specific siRNA or siRNA:*Mll1* and treated with 50 ng/ml Wnt3a for the indicated time points. Co-localisation of β-catenin and DAPI was measured within the brightfield image of the cell using the ImageStream^X^ imaging flow cytometer. Fold change Rd. median versus WT unstimulated values were calculated using Pearson's correlation coefficient and Fisher's discriminant ratio for β-catenin and DAPI localisation within the cell. Bars represent β-catenin and DAPI co-localisation when compared to the WT unstimulated cells. A Student's *t*-test was used to assess significance of differences between samples (N = 3). * indicates significant differences versus WT control (p < 0.05). # indicates significant differences versus genotype specific control (p < 0.05). (A-B) RNA interference knockdowns were performed with two independent siRNAs to *Mll1*. (C) Representative images of β-catenin and DAPI localisation measured using ImageStream^X^. (D) Nuclear translocation of β-catenin in response to Wnt3a was confirmed by western blot with a pan-β-catenin antibody in WT and *Cks*-deficient MEFs. (E) Measurement of PI3K/Akt pathway activation in whole cell lysates by Akt versus phospho-Akt western blots, in WT and *Cks*-deficient MEFs, after Wnt3a treatment for the indicated time points. Actin and Histone H3 were probed for as whole cell fraction and nuclear fraction controls. Western blots are representative of 3 independent experiments.

A subset of Mll1 target genes (e.g. *Sfrp1*, *Rspo2*), which are significantly altered in *Cks*-deficient MEFs (Fig. 1D) are key players in the Wnt signalling pathway. To study Wnt signalling in *Cks*-deficient MEFs, we used the TOPFlash reporter assay for β-catenin-induced T-cell factor/lymphoid-enhancing factor (TCF/LEF) transcriptional activity in response to Wnt3a, a Wnt ligand commonly used for in vitro studies [43]. This revealed a significant contribution of Cks proteins to TCF/LEF transcriptional activity (Fig. 2A). Indeed, *Cks1*^*−/−*^ MEFs displayed a significantly reduced basal activity compared to WT (p = 0.01), and were unresponsive to Wnt3a treatment (versus WT p < 0.05). Conversely, *Cks2*^*−/−*^ MEFs had a similar basal activity level compared to the WT control, but showed significantly faster induction kinetics and reached greater maximal TCF/LEF activity in response to Wnt3a (p < 0.01; Fig. 2A). This demonstrates a complex contribution of the Cks1/Cks2 axis to Wnt signalling, potentially occurring through Mll1 protein regulation.

To investigate whether the different Mll1 levels observed in *Cks*-deficient MEFs were responsible for the opposing effects of Cks1 and Cks2 on Wnt signalling, the same experiment was repeated in MEFs after manipulation of *Mll1* expression (Fig. S2). Overexpression of *MLL1* reduced the induction kinetics and maximal response of TCF/LEF activity in response to Wnt3a in WT (p < 0.02 at 16 h and 32 h) and more strikingly in *Cks2*^*−/−*^ (p < 0.01 at 16 h and 32 h; Fig. 2A +* MLL1*) MEFs. This is consistent with the possibility that reduced Mll1 levels in *Cks2*^*−/−*^ MEFs contributed to increased Wnt signalling. As expected, since *Cks1*^*−/−*^ MEFs already have a greater level of Mll1 compared to WT and *Cks2*^*−/−*^ MEFs, *Cks1*^*−/−*^ cells were unresponsive to MLL1 overexpression, and consequently are unable to further repress Wnt signalling activity. Conversely, siRNA-mediated knockdown of *Mll1* in WT and *Cks1*^*−/−*^ cells increased the basal TCF/LEF activity, and the kinetic of response by Wnt3a, to a small but significant and reproducible degree (Fig. 2A siRNA:*Mll1*). In *Cks2*^*−/−*^ cells, *Mll1* knockdown also significantly increased sensitivity to Wnt3a (Fig. 2A siRNA:*Mll1*). These effects were specific to Wnt signalling, as no activation was observed with the FOPFlash negative control construct (data not shown). The partial restoration of Wnt signalling in *Cks1*^*−/−*^ MEFs by *Mll1* knockdown again suggests that Mll1 is a target of Cks proteins, which impacts on transcriptional regulation and downstream signalling pathways. Thus, the respective impacts of *Cks1* or *Cks2* knockout were partially reversed by readjusting Mll1 protein levels.

A key step during Wnt signal transduction is the translocation of β-catenin from the cytoplasm to the nucleus [44]. To investigate this process, we used an imaging flow cytometer to measure the co-localisation of β-catenin with a nuclear dye [45] (Amnis ImageStream^X^ Mk II; Fig. S3). As expected, nuclear β-catenin was significantly increased in WT MEFs in response to Wnt3a treatment (p < 0.05; Fig. 2B-C and Fig. S4). Nuclear translocation of β-catenin in *Cks1*^*−/−*^ cells was significantly reduced compared to WT (p < 0.05), with lower basal nuclear β-catenin and minimal nuclear translocation in response to Wnt3a. In contrast, *Cks2*^*−/−*^ MEFs had a significantly higher basal level of nuclear β-catenin compared to WT (p < 0.05), and were minimally responsive to Wnt3a, indicating that increased TCF/LEF activity is partially due to constitutively high nuclear β-catenin (Fig. 2B). Cell fractionation followed by western blot analysis confirmed decreased nuclear localisation of β-catenin in *Cks1*^*−/−*^ MEFs, and proportionally higher nuclear β-catenin in *Cks2*^*−/−*^ MEFs when compared to WT MEFs (Fig. 2D). As an additional readout for Wnt signalling, we analysed Akt phosphorylation. Wnt3a treatment caused Akt phosphorylation to be maintained at its basal level in *Cks1*^*−/−*^, and to be markedly upregulated in *Cks2*^*−/−*^ MEFs when compared to WT control (Fig. 2E).

Taken together, these data demonstrate that opposing effects of Cks1 and Cks2 on Mll1 protein levels have a significant impact on cellular Wnt signalling in MEFs.

### 3.3 Aberrant *CKS* expression in *MLL*-rearranged leukaemias

**Fig. 3:**
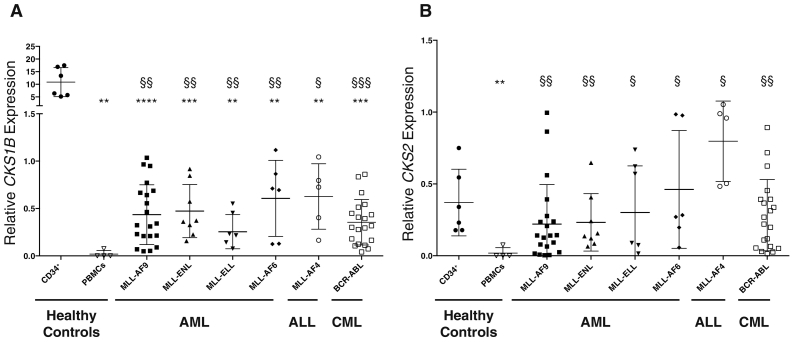
*CKS1B* and *CKS2* expression is higher in diagnostic bone marrow samples from patients carrying *MLL*-translocations compared to PBMCs from healthy controls. RNA extracted from human umbilical cord blood CD34^+^ cells, PBMCs from healthy donors, and leukaemic patient samples, were assessed for (A) *CKS1B* and (B) *CKS2* expression by TaqMan quantitative PCR. Three independent control genes were used (*ABL*, *GUS*, *B2M*) to measure relative RNA abundance. Values are represented as relative gene expression versus *GUS* control. Each point represents one patient. Median bars with standard deviation are presented for each set of samples. A Student's *t*-test was used to assess significance, and * indicates significance versus CD34^+^ cells, and § indicates significance versus PBMCs.

Expression of *CKS1B* and *CKS2* was investigated by RT-qPCR in a total of 65 patients diagnosed with AML, ALL or CML (Fig. 3A-B). CD34 enriched cord blood mononuclear cells (CD34^+^) and peripheral blood mononuclear cells (PBMCs) from healthy donors were used as controls. *CKS1B* and *CKS2* showed significantly increased expression in patient samples carrying *MLL-AF9* (n = 20, *CKS1B* p = 0.0001, *CKS2* p = 0.004), *MLL-ENL* (n = 8, *CKS1B* p = 0.0024, *CKS2* p = 0.0194), *MLL-AF6* (n = 6, *CKS1B* p = 0.0155, *CKS2* p = 0.0456), *MLL-AF4* (n = 5, *CKS1B* p = 0.0165, *CKS2* p = 0.0031) and *BCR-ABL* (n = 20, *CKS1B* p = 0.0001, *CKS2* p = 0.0002) fusions compared to PBMCs. *CKS1B*, but not *CKS2*, exhibited significantly higher expression in patient samples carrying the *MLL-ELL* fusion (n = 6, *CKS1B* p = 0.0128) compared to PBMCs (Fig. 3A-B). Whereas *CKS2* expression in all leukaemic cells tested was similar to CD34^+^ cells, *CKS1B* was expressed at significantly higher levels in CD34^+^ cells than leukaemic patient samples. These data are in agreement with publicly available datasets, including the MILE study [46], which also found significantly higher *CKS1B* and *CKS2* expression in healthy bone marrow mononuclear cells compared to bulk *MLLr* leukaemic samples. Altogether, these analyses revealed a dysregulation of *CKS* expression in *MLLr* leukaemia, but also in *BCR-ABL* positive CML [47], consistent with putative roles for *CKS1B* and *CKS2* in leukaemogenesis. *CKS* expression levels might also be linked to the relative stage of maturation in normal haematopoiesis (CD34^+^ versus PBMC).

### 3.4 Heterogeneous associations of MLL-FPs with CKS proteins

To determine whether CKS proteins interact with MLL-FPs, co-immunoprecipitation assays with antibodies specific to the MLL-FP (or an IgG isotype control as a negative control) were performed. Using cell lines carrying *MLL-AF9* (THP-1 – myeloid lineage), *MLL-AF6* (ML-2 – myeloid lineage) and *MLL-ENL* (KOPN-8 – lymphoid lineage) translocations, the fusion partners were pulled down and western blotted for endogenous CKS1 and CKS2 (Fig. S5). All fusion partner specific antibodies were able to immunoprecipitate the endogenous MLL-FPs, whereas IgG controls were not. When probing for CKS1 and CKS2, the MLL-AF9 fusion protein was found to retain interactions with both CKS1 and CKS2 (Fig. S5A). MLL-AF6 retained interaction only with CKS1 (Fig. S5B), while no interaction could be detected between MLL-ENL and either CKS1 or CKS2 (Fig. S5C). These results demonstrate that MLL interaction with CKS proteins is affected by oncogenic fusion partners in various ways, reinforcing the concept that MLL-FPs shape distinct protein complexes [28], [48].

### 3.5 MLL fusion partners dictate the roles of CKS proteins in Wnt signalling

**Fig. 4:**
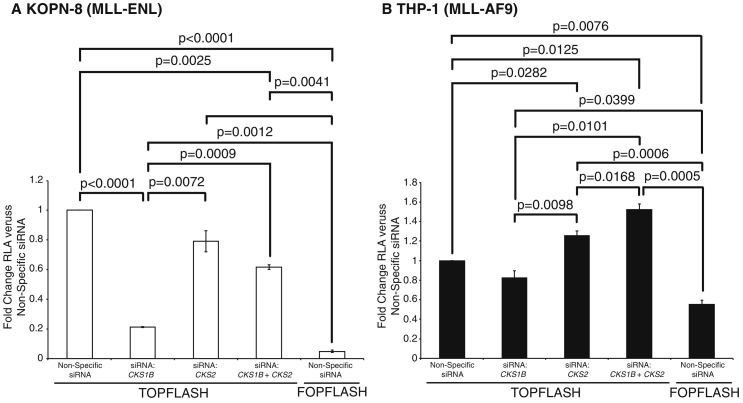
Modulation of Wnt signalling in *MLL*-translocation cell lines by CKS1 and CKS2. TCF/LEF activity was measured in (A) KOPN-8 (MLL-ENL) and (B) THP-1 (MLL-AF9) cell lines using the TOPFlash reporter plasmid (or FOPFlash plasmid control) and pRL-TK Renilla control. Cell lines were co-transfected with siRNA to knockdown expression of *CKS1B*, *CKS2* or *CKS1B* + *CKS2*. Values are represented as fold change RLA versus Non-specific siRNA treated cells. Significance was tested using the Student's *t*-test (N = 3).

Active Wnt signalling is required for the development of leukaemic stem cells, disease progression, and, in some cases, loss of sensitivity to chemotherapeutic agents [49], [50], [51], [52]. To explore CKS1 and CKS2 Wnt signalling contributions in *MLL*-translocation carrying cell lines, we again used the TOPFlash assay. In agreement with published data, we found the Wnt signalling pathway to be highly active in KOPN-8 cells [53] and active, albeit to a lesser extent, in THP-1 cells [54] (Fig. S6A). *CKS1B* knockdown significantly reduced Wnt signalling in KOPN-8 cells, but had no effect on THP-1 cells (Fig. 4A-B and Figs. S6B-C and S7). Conversely, *CKS2* knockdown significantly increased Wnt signalling in THP-1 cells, but had no effect on KOPN-8 cells (Fig. 4A-B). *CKS1B* and *CKS2* double knockdown produced an intermediate phenotype in KOPN-8 cells, but interestingly, further increased Wnt signalling in THP-1 cells (Fig. 4A-B). It should be noted that RNA interference targeting *CKS1B*, *CKS2*, or a combination of both siRNAs did not compromise the cell viability or proliferation of THP-1 or KOPN-8 cells (data not shown). Considering that *CKS* knockdowns were not highly efficient in THP-1 and KOPN-8 cells (Fig. S7), these data show the sensitivity to the CKS1/CKS2 axis on the regulation of MLL, and consequently Wnt signalling in *MLL*-translocation lines.

### 3.6 Inhibition of CKS-dependent protein degradation is cytotoxic for *MLL*-translocation lines

**Fig. 5:**
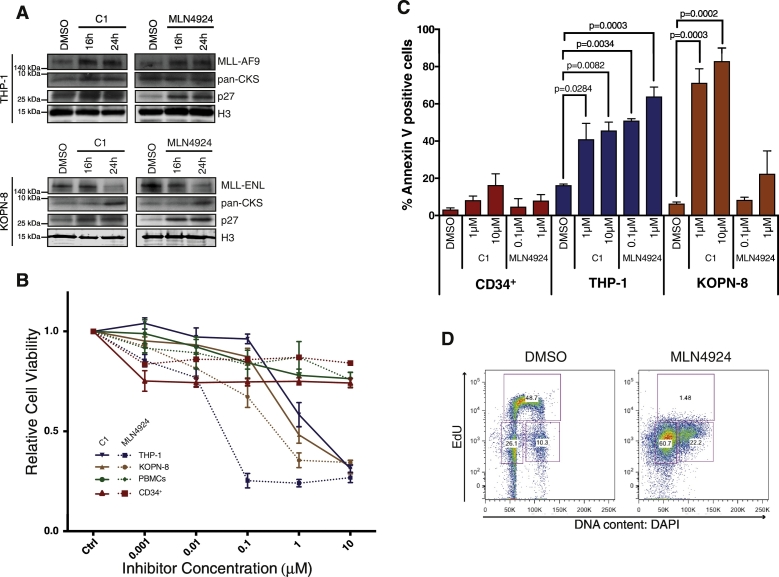
*MLL*-translocation cell lines are sensitive to inhibition of pan-Cullin-dependent and CKS1-dependent protein degradation. (A) Western blots for THP-1 (*MLL-AF9*) and KOPN-8 (*MLL-ENL*) cells treated with vehicle control (DMSO), 0.1 μM MLN4924 or 1 μM C1 for 16 h and 24 h. Histone H3 was used as a loading control. Western blots are representative of 3 independent experiments. (B) Cell viability was assessed for THP-1, KOPN-8, healthy PBMC and CD34^+^ control cells after 3 days treatment with indicated concentrations of the pan-Cullin inhibitor (MLN4924) or the SKP2-CKS1 inhibitor (C1). Results represent the mean of 3 independent experiments with standard deviation bars. (C) Proportion of apoptotic CD34^+^, THP-1 and KOPN-8 cells, in response to two different concentrations of C1 or MLN4924, was measured by PI and Annexin V-FITC staining using flow cytometry. Significance was tested using the Student's *t*-test versus DMSO treated cells (N = 3). (D) Cell cycle status was measured for KOPN-8 cells in response to DMSO vehicle control or MLN4924 (1 μM) using the Click-iT EdU incorporation kit. Graphs are representative of 3 independent experiments.

We next investigated the effect of small molecule inhibitors targeting the Cullin Ring Ligase (CRL) family of protein degradation machinery, of which CKS1 acts as a constituent. For this purpose, we used the small molecule inhibitor of the NEDD-8 activating enzyme (NAE), MLN4924 (or Pevonedistat), which has shown promise for targeting of protein degradation in cancer therapy, including diffuse large B-cell lymphoma and AML [55], [39], [56]. We also tested C1, a small molecule inhibitor of the SKP2-CKS1 degradation complex [57]. These inhibitors have both been shown to reduce p27 ubiquitination, with implications for cancer treatment [39], [57], [58], [59], [60]. Whereas NAE inhibitors stabilise a broad range of Cullin-RING-based ubiquitin ligase targets (e.g. pIκBα, CDT-1, p27, p21) [55], [39], [61], SKP2-CKS1 inhibitors exclusively suppress degradation of CKS1-dependent SKP2 targets (e.g. p27), but not of CKS1-independent SKP2 targets (e.g. p21) [57], [58]. MLN4924 treatment of THP-1 and KOPN-8 cells resulted in a large increase in p27 protein level, as previously described in a different MLL-AF9 cell line [39], confirming inhibition of the Cullin-containing SCF E3 ligase (Fig. 5A). In agreement with a previous study in melanoma cells [57], treatment with the SKP2-CKS1 inhibitor C1 increased p27 protein levels in both cell lines. Interestingly, inhibitor treatments had opposite effects on MLL-fusion protein expression levels. Indeed, while KOPN-8 cells exhibited a decrease of MLL-ENL protein expression after MLN4924 or C1 treatment, MLL-AF9 protein level increased in THP-1 cells under these two conditions (Fig. 5A). CKS protein levels were also affected; there was an increase in total CKS protein in all cases, except in THP-1 cells treated with MLN4924 (Fig. 5A).

When THP-1 and KOPN-8 cells were challenged with C1 or MLN4924, both cell lines showed significant reductions in cell viability compared to cells treated with DMSO (vehicle), or CD34^+^ and PBMCs from healthy donors (Fig. 5B). These results appear to contradict those obtained via RNA interference (i.e. showing no variation in cell viability), but this discrepancy is most likely explained by the incomplete knockdown, as previously stated (Fig. S7). To confirm the drug sensitivity of these cell lines when compared to healthy controls, clonogenic assays were performed. Colony formation of *MLLr* AML lines was reduced following C1 or MLN4924 treatment, compared to the vehicle control, whereas CD34^+^ colony formation remained unaffected (Fig. S8). The reduction in THP-1 cell viability correlated with a significant increase in apoptotic cells (Annexin V positive; Fig. 5C) and activation of Caspase 3 (Fig. S9) for both C1 and MLN4924. Surprisingly, while the KOPN-8 cells showed increased apoptosis after C1 treatment, MLN4924 caused cell cycle-arrest in G1 or G2 phase, devoid of cells progressing through S-phase (Fig. 5D and Fig. S9). We tested two additional cell lines: ML-1, an AML cell line expressing MLL-AF6, and RS4;11, an ALL cell line expressing MLL-AF4. Interestingly, both responded to the inhibitor C1, whereas MNL4924 treatment induced cell death only in the ML-1 cell line (Fig. S10). Thus, effective targeting of the neddylation pathway using MLN4924 seems to be restricted to *MLLr* AML lines, while both *MLLr* AML and *MLLr* ALL lines appear to be sensitive to C1 at doses which exhibit low toxicity in healthy cells.

Altogether, these results indicate that Cullin-based protein degradation is important for *MLL*-translocation cell viability, and a range of SKP2-CKS1 targets, the MLL-FPs themselves included, may be involved in fine-tuning this process.

## 4 Discussion

Previous studies, describing the requirement of Cks1 [8], [47] and Cks2 [8], [13] in cell cycle dynamics, have postulated that targets of the Cks1/Cks2 axis may extend beyond the specific subset of Cyclins and CKIs [5], [7], [10], [13], [62]. Here, we provide the first evidence for an essential role of the Cks1/Cks2 axis in the regulation of Mll1 expression. The divergent roles Cks1 and Cks2 play in Mll1 stability have major downstream consequences on the Wnt signalling pathway. We also identified an impact on *MLLr* cell viability when interfering with the CKS1/CKS2 axis.

We demonstrated the requirement of Cks1 and Cks2 in Mll1 protein expression throughout the cell cycle. Interestingly, knockout of *Cks1* mimics the stable Mll1 protein levels previously described in leukaemia [30], while knockout of *Cks2* has the opposite effect, with Mll1 protein levels almost undetectable. The downstream effect of MLL1 stabilisation (as observed in *Cks1*^*−/−*^ MEFs) has been well documented in *MLLr* leukaemia [28], [30], [31], [63], [64], in which the normal epigenetic profile and cooperating signalling pathways, governed by *MLL1*, are hijacked by MLL-FPs [48], [65], [66], [67], [68], [69]. One key downstream pathway is the Wnt/β-catenin pathway, which is involved in both primary mixed-lineage leukaemia development [45], [49], [50] and drug resistance [70], [71]. Whilst the bone marrow niche is the primary provider of extracellular Wnt signals required for both haematopoietic stem cell and leukaemic stem cell self-renewal [51], [72], [73], [74], MLL1 and MLL-FPs play critical roles in intracellular regulation of Wnt signalling [52], [75], [76], [77]. Therefore, the Mll1-dependent impact of the Cks1/Cks2 axis on Wnt signalling is of particular interest. The Wnt signalling changes that we observed in *Cks*-deficient MEFs are consistent with those reported in both the *Skp2*^*−/−*^ [74] and *p27*^*−/−*^ [78], [79], [80] mouse models, indicating that Cks1/Cks2/SCF^Skp2^ dynamics are likely responsible for these alterations. Interestingly, *CKS1B* (*Myc*-dependent) [14] and *CKS2* [81] expression can also be regulated by the Wnt signalling effector β-catenin, forming a feedback loop mediated through the CKS1/CKS2 axis, which may be required to balance intracellular Wnt signalling, in response to both internal and external stimuli. In cases in which Wnt signalling is responsible for the emergence of leukaemic stem cells that are resistant to key therapeutic agents [49], [70], [71], the regulation of Mll1, and consequently Wnt signalling, by the CKS1/CKS2 axis provides a positive new alternative for combating these leukaemic stem cells and keeping Wnt signalling under control.

Overexpression of *CKS1B* and *CKS2* has been documented in a variety of cancers [6], [16], [18], [19], [20], [21], [22], [23], [82], downstream of a variety of oncogenes, and their high expression has been correlated with poor prognosis. Similarly, *CKS1B* expression has been reported to be high in CML patients at blast crisis and, further to this, significantly reduced after imatinib (STI-571) treatment [47]. Investigation of Cks1 roles in the Eμ-Myc lymphoma mouse model indicates that Cks1 functions in cancer expand beyond p27 regulation, and other pathways may be regulated by Cks1 [14]. We found that *MLLr* leukaemic blasts have upregulation of *CKS1B* and *CKS2* compared to terminally differentiated cells (PBMCs), and further increase *CKS1B* expression in CD34^+^ cells, a finding further confirmed in the MILE study [46]. This suggests that *CKS*-upregulation could be a significant factor in these cancer cells. Higher expression of *CKS1B* and *CKS2* in leukaemic cells, compared to terminally differentiated cells, may be an important part of the epigenetic re-programming of normal blood cells to block differentiation and increase the number of more stem-like leukaemic cells by MLL-FPs [28], [64], [83], [84], [85]. Furthermore, high levels of *CKS1B* observed in haematopoietic stem and progenitor cells (CD34^+^) indicate that *CKS1B* may be a central factor of the stem cell program hijacked by MLL-FPs in *MLLr* leukaemia.

The variety of leukaemogenic MLL-FPs reported produce a heterogeneous disease, dependent on the FP present [28], [86]. This heterogeneity was reflected in the presence or absence of interactions with CKS1 and/or CKS2, depending on the respective MLL-FPs that we investigated. Despite a lack of interaction between CKS1/2 and MLL-ENL, Wnt signalling was still affected in MLL-ENL cells following *CKS1* and/or *CKS2* knockdown. A probable explanation is that CKS1/2 are displaced in the larger COMPASS-like complex [28] built by MLL-ENL, which might impact on additional downstream functions. Although Cks1/2 was shown to interact with MII1 (N- and C-terminal subunits), interaction between CKS1/2 and the fusion partner or the wild-type copy of the fusion partner cannot be excluded. Further to this, stabilisation of the WT MLL1 protein in MLL-FP cells may play a crucial role in combating aberrant MLL-FP epigenetic functions [36]. Disrupting MLL-FP transcriptional complexes is perceived to be a promising route for eradicating aggressive mixed-lineage leukaemias [28], [37], [63], and our discovery of new MLL-FP complex interactions may provide new druggable targets.

As previously reported for p27 [8], Cks1 constrains Mll1 function by promoting its degradation, while Cks2 stabilises Mll1. The fact that both *CKS1* and *CKS2* are often overexpressed in primary cancers and tumour-derived cell lines suggests that neoplastic cell proliferation depends on a carefully balanced signalling network in which both proteins partake. Therefore, the promise of ubiquitin ligase inhibitors in combating tumour cell growth can be rationalised by their impact on this signalling network, such that it no longer supports uncontrolled cell proliferation.

The clinical utility of inhibiting protein degradation in leukaemia, and more specifically the Cullin-RING ligase family, is currently the focus of clinical trials (NCT00911066 and NCT01814826). Use of the NAE inhibitor MLN4924 indicates that inhibition of Cullin-RING ligases is effective in targeting mixed-lineage leukaemia [60], [87], however, resistant cells arise upon treatment [88]. The SCF^Skp2-Cks1^ complex is one constituent of the Cullin-RING ligase family, specifically targeting a small range of proteins within the larger range of pan-Cullin mediated degradation [89], [90], [91]. The new generation of inhibitors targeting the SCF^SKP2-CKS1^ complex provides a more specific and sensitive treatment, which may reduce off-target effects [57], [58], [92]. Currently, therapies targeting epigenetic functions of MLL-FPs, including BET [70], [71], [93] and DOT1L [94] inhibitors, produce satisfactory results initially, but resistance emerges due to transcriptional plasticity [70], [71], [95], [96]. Our data suggest that CKS-inhibiting agents, in combination therapy, might be able to eradicate both quiescent leukaemic stem cells, and resistant proliferating clones. Indeed, while it is anticipated that p27 modulation, promoting cell death, would be similar in all leukaemic cells, the dysregulation of the Wnt pathway may be more pronounced in vivo. The Wnt pathway is crucial for various leukaemic stem cell (LSC) features involved in LSC drug resistance (e.g. survival and tumour-niche communication), indicating that inhibiting CKS proteins may have a dual purpose in leukaemic survival, targeting both p27 and Wnt signalling.

The emergence of new inhibitors targeting alternative functions of the CKS1/CKS2 axis [97] provides great promise. Future studies will be needed to fully understand the molecular dynamics and downstream functions of the CKS1/CKS2/SKP2 pathway as new players are still being identified [98], [99], and in vivo studies will be required to validate their therapeutic potential.

## Conflict of interest

Thomas A. Milne is one of the founding shareholders of Oxstem Oncology (OSO), a subsidiary company of OxStem Ltd. (2016). The other authors declare no conflict of interest.

## Transparency document

Transparency document. Image 2

## Supplementary data

Supplementary material Image 1

## References

[1] Reynard G.J., Reynolds W., Verma R., Deshaies R.J. (2000). Cks1 is required for G(1) cyclin-cyclin-dependent kinase activity in budding yeast. Mol. Cell. Biol..

[2] McGrath D.A., Balog E.R.M., Kõivomägi M., Lucena R., Mai M.V., Hirschi A., Kellogg D.R., Loog M., Rubin S.M. (2013). Cks confers specificity to phosphorylation-dependent CDK signaling pathways. Nat. Struct. Mol. Biol..

[3] Morris M.C., Kaiser P., Rudyak S., Baskerville C., Watson M.H., Reed S.I. (2003). Cks1-dependent proteasome recruitment and activation of CDC20 transcription in budding yeast. Nature.

[4] Yu V.P.C.C., Baskerville C., Gronenfelder B., Reed S.I. (2005). A kinase-independent function of Cks1 and Cdk1 in regulation of transcription. Mol. Cell.

[5] Martinsson-Ahlzen H.S., Liberal V., Grunenfelder B., Chaves S.R., Spruck C.H., Reed S.I., Martinsson-Ahlzén H.-S., Liberal V., Grünenfelder B., Chaves S.R., Spruck C.H., Reed S.I. (2008). Cyclin-dependent kinase-associated proteins Cks1 and Cks2 are essential during early embryogenesis and for cell cycle progression in somatic cells. Mol. Cell. Biol..

[6] Westbrook L., Manuvakhova M., Kern F.G., Estes N.R., Ramanathan H.N., Thottassery J.V., Estes N.R., Ramanathan H.N., Thottassery J.V. (2007). Cks1 regulates cdk1 expression: a novel role during mitotic entry in breast cancer cells. Cancer Res..

[7] Spruck C., Strohmaier H., Watson M., Smith A.P.L., Ryan A., Krek W., Reed S.I. (2001). A CDK-independent function of mammalian Cks1: targeting of SCFSkp2 to the CDK inhibitor p27Kip1. Mol. Cell.

[8] Frontini M., Kukalev A., Leo E., Ng Y.-M.M., Cervantes M., Cheng C.-W.W., Holic R., Dormann D., Tse E., Pommier Y., Yu V.P.C.C. (2012). The CDK subunit CKS2 counteracts CKS1 to control Cyclin A/CDK2 activity in maintaining replicative fidelity and neurodevelopment. Dev. Cell.

[9] Radulovic M., Crane E., Crawford M., Godovac-Zimmermann J., Yu V.P.C.C. (2010). CKS proteins protect mitochondrial genome integrity by interacting with mitochondrial single-stranded DNA-binding protein. Mol. Cell. Proteomics.

[10] Liberal V., Martinsson-Ahlzén H.-S., Liberal J., Spruck C.H., Widschwendter M., McGowan C.H., Reed S.I., Martinsson-Ahlzen H.S., Liberal J., Spruck C.H., Widschwendter M., McGowan C.H., Reed S.I. (2012). Cyclin-dependent kinase subunit (Cks) 1 or Cks2 overexpression overrides the DNA damage response barrier triggered by activated oncoproteins. Proc. Natl. Acad. Sci. U. S. A..

[11] Lin L., Fang Z., Lin H., You H., Wang J., Su Y., Wang F., Zhang Z.Y. (2016). Depletion of Cks1 and Cks2 expression compromises cell proliferation and enhance chemotherapy-induced apoptosis in HepG2 cells. Oncol. Rep..

[12] Ganoth D., Bornstein G., Ko T.K., Larsen B., Tyers M., Pagano M., Hershko A. (2001). The cell cycle regulatory protein Cks1 is required for SCFSkp2 mediated ubiquitinylation of p27. Nat. Cell Biol..

[13] Spruck C.H., de Miguel M.P., Smith A.P.L., Ryan A., Stein P., Schultz R.M., Lincoln A.J., Donovan P.J., Reed S.I. (2003). Requirement of Cks2 for the first metaphase/anaphase transition of mammalian meiosis. Science.

[14] Keller U.B., Old J.B., Dorsey F.C., Nilsson J.A., Nilsson L., MacLean K.H., Chung L., Yang C., Spruck C.H., Boyd K., Reed S.I., Cleveland J.L. (2007). Myc targets Cks1 to provoke the suppression of p27kip1, proliferation and lymphomagenesis. EMBO J..

[15] Bhatt K.V., Hu R., Spofford L.S., Aplin A.E. (2007). Mutant B-RAF signaling and cyclin D1 regulate Cks1/S-phase kinase-associated protein 2-mediated degradation of p27Kip1 in human melanoma cells. Oncogene.

[16] Chang H., Qi X., Trieu Y., Xu W., Reader J.C., Ning Y., Reece D. (2006). Multiple myeloma patients with CKS1B gene amplification have a shorter progression-free survival post-autologous stem cell transplantation. Br. J. Haematol..

[17] Liu Z., Fu Q., Lv J., Wang F., Ding K. (2008). Prognostic implication of p27Kip1, Skp2 and Cks1 expression in renal cell carcinoma: a tissue microarray study. J. Exp. Clin. Cancer Res..

[18] Kang M.A., Kim J.H.J.-T.H.T., Kim J.H.J.-T.H.T., Kim S.-Y.Y., Kim Y.H., Il Yeom Y., Lee Y., Lee H.G. (2009). Upregulation of the cycline kinase subunit CKS2 increases cell proliferation rate in gastric cancer. J. Cancer Res. Clin. Oncol..

[19] Shen D.-Y.Y., Fang Z.-X.X., You P., Liu P.-G.G., Wang F., Huang C.-L.L., Yao X.-B.B., Chen Z.-X.X., Zhang Z.-Y.Y. (2010). Clinical significance and expression of cyclin kinase subunits 1 and 2 in hepatocellular carcinoma. Liver Int..

[20] Zhan F., Colla S., Wu X., Chen B., Stewart J.P., Kuehl W.M., Barlogie B., Shaughnessy J.D. (2007). CKS1B, overexpressed in aggressive disease, regulates multiple myeloma growth and survival through SKP2- and p27Kip1-dependent and -independent mechanisms. Blood.

[21] Huang J., Zhou Y., Thomas G.S., Gu Z., Yang Y., Xu H., Tricot G., Zhan F. (2015). NEDD8 inhibition overcomes CKS1B-induced drug resistance by upregulation of p21 in multiple myeloma. Clin. Cancer Res..

[22] Wang X.-C., Tian L.-L., Tian J., Wu H.-L., Meng A.-M. (2009). Overexpression of Cks1 is associated with poor survival by inhibiting apoptosis in breast cancer. J. Cancer Res. Clin. Oncol..

[23] Wang X.-C.C., Tian J., Tian L.-L.L., Wu H.-L.L., Meng A.M., Ma T.-H.H., Xiao J., Xiao X.-L.L., Li C.-H.H. (2009). Role of Cks1 amplification and overexpression in breast cancer. Biochem. Biophys. Res. Commun..

[24] Milne T.A., Briggs S.D., Brock H.W., Martin M.E., Gibbs D., Allis C.D., Hess J.L. (2002). MLL targets SET domain methyltransferase activity to Hox gene promoters. Mol. Cell.

[25] Milne T.A., Hughes C.M., Lloyd R., Yang Z., Rozenblatt-Rosen O., Dou Y., Schnepp R.W., Krankel C., Livolsi V.A., Gibbs D., Hua X., Roeder R.G., Meyerson M., Hess J.L. (2005). Menin and MLL cooperatively regulate expression of cyclin-dependent kinase inhibitors. Proc. Natl. Acad. Sci. U. S. A..

[26] Wang P., Lin C., Smith E.R., Guo H., Sanderson B.W., Wu M., Gogol M., Alexander T., Seidel C., Wiedemann L.M., Ge K., Krumlauf R., Shilatifard A. (2009). Global analysis of H3K4 methylation defines MLL family member targets and points to a role for MLL1-mediated H3K4 methylation in the regulation of transcriptional initiation by RNA polymerase II. Mol. Cell. Biol..

[27] Yokoyama A., Kitabayashi I., Ayton P.M., Cleary M.L., Ohki M. (2002). Leukemia proto-oncoprotein MLL is proteolytically processed into 2 fragments with opposite transcriptional properties. Blood.

[28] Ballabio E., Milne T.A. (2012). Molecular and epigenetic mechanisms of MLL in human leukemogenesis. Cancer.

[29] Yokoyama A., Ficara F., Murphy M.J., Meisel C., Hatanaka C., Kitabayashi I., Cleary M.L. (2013). MLL becomes functional through intra-molecular interaction not by proteolytic processing. PLoS One.

[30] Liu H., Cheng E.H., Hsieh J.J. (2007). Bimodal degradation of MLL by SCFSkp2 and APCCdc20 assures cell cycle execution: a critical regulatory circuit lost in leukemogenic MLL fusions. Genes Dev..

[31] Li B.E., Ernst P. (2014). Two decades of leukemia oncoprotein epistasis: the MLL1 paradigm for epigenetic deregulation in leukemia. Exp. Hematol..

[32] Daser A., Rabbitts T.H. (2004). Extending the repertoire of the mixed-lineage leukemia gene MLL in leukemogenesis. Genes Dev..

[33] Hess J.L. (2004). MLL: a histone methyltransferase disrupted in leukemia. Trends Mol. Med..

[34] Meyer C., Kowarz E., Hofmann J., Renneville A., Zuna J., Trka J., Ben Abdelali R., Macintyre E., De Braekeleer E., De Braekeleer M., Delabesse E., de Oliveira M.P., Cavé H., Clappier E., van Dongen J.J.M., Balgobind B.V., van den Heuvel-Eibrink M.M., Beverloo H.B., Panzer-Grümayer R., Teigler-Schlegel A., Harbott J., Kjeldsen E., Schnittger S., Koehl U., Gruhn B., Heidenreich O., Chan L.C., Yip S.F., Krzywinski M., Eckert C., Möricke A., Schrappe M., Alonso C.N., Schäfer B.W., Krauter J., Lee D.A., Zur Stadt U., Te Kronnie G., Sutton R., Izraeli S., Trakhtenbrot L., Lo Nigro L., Tsaur G., Fechina L., Szczepanski T., Strehl S., Ilencikova D., Molkentin M., Burmeister T., Dingermann T., Klingebiel T., Marschalek R. (2009). New insights to the MLL recombinome of acute leukemias. Leukemia.

[35] Marschalek R. (2016). Systematic classification of mixed-lineage leukemia fusion partners predicts additional cancer pathways. Ann. Lab. Med..

[36] Liang K., Volk A.G., Haug J.S., Marshall S.A., Woodfin A.R., Bartom E.T., Gilmore J.M., Florens L., Washburn M.P., Sullivan K.D., Espinosa J.M., Cannova J., Zhang J., Smith E.R., Crispino J.D., Shilatifard A. (2017). Therapeutic targeting of MLL degradation pathways in MLL-rearranged leukemia. Cell.

[37] Neff T., Armstrong S.A. (2013). Recent progress toward epigenetic therapies: the example of mixed lineage leukemia. Blood.

[38] Knorr K.L.B., Finn L.E., Smith B.D., Hess A.D., Foran J.M., Karp J.E., Kaufmann S.H. (2017). Assessment of drug sensitivity in hematopoietic stem and progenitor cells from acute myelogenous leukemia and myelodysplastic syndrome ex vivo. Stem Cells Transl. Med..

[39] Swords R.T., Kelly K.R., Smith P.G., Garnsey J.J., Mahalingam D., Medina E., Oberheu K., Padmanabhan S., Dwyer M.O., Nawrocki S.T., Giles F.J., Carew J.S., O'Dwyer M., Nawrocki S.T., Giles F.J., Carew J.S. (2010). Inhibition of NEDD8-activating enzyme: a novel approach for the treatment of acute myeloid leukemia. Blood.

[40] Beillard E., Pallisgaard N., van der Velden V.H.J., Bi W., Dee R., van der Schoot E., Delabesse E., Macintyre E., Gottardi E., Saglio G., Watzinger F., Lion T., van Dongen J.J.M., Hokland P., Gabert J. (2003). Evaluation of candidate control genes for diagnosis and residual disease detection in leukemic patients using “real-time” quantitative reverse-transcriptase polymerase chain reaction (RQ-PCR) - a Europe against cancer program. Leukemia.

[41] George T.C., Fanning S.L., Fitzgerald-Bocarsly P., Fitzgeral-Bocarsly P., Medeiros R.B., Highfill S., Shimizu Y., Hall B.E., Frost K., Basiji D., Ortyn W.E., Morrissey P.J., Lynch D.H. (2006). Quantitative measurement of nuclear translocation events using similarity analysis of multispectral cellular images obtained in flow. J. Immunol. Methods.

[42] Dinkel H., Van Roey K., Michael S., Davey N.E., Weatheritt R.J., Born D., Speck T., Krüger D., Grebnev G., Kuban M., Strumillo M., Uyar B., Budd A., Altenberg B., Seiler M., Chemes L.B., Glavina J., Sánchez I.E., Diella F., Gibson T.J. (2014). The eukaryotic linear motif resource ELM: 10 years and counting. Nucleic Acids Res..

[43] Veeman M.T., Slusarski D.C., Kaykas A., Louie S.H., Moon R.T. (2003). Zebrafish prickle, a modulator of noncanonical Wnt/Fz signaling, regulates gastrulation movements. Curr. Biol..

[44] Willert K., Nusse R. (1998). Beta-catenin: a key mediator of Wnt signaling. Curr. Opin. Genet. Dev..

[45] Gandillet A., Park S., Lassailly F., Griessinger E., Vargaftig J., Filby A., Lister T.A., Bonnet D. (2011). Heterogeneous sensitivity of human acute myeloid leukemia to β-catenin down-modulation. Leukemia.

[46] Haferlach T., Kohlmann A., Wieczorek L., Basso G., Te Kronnie G., Béné M.-C., De Vos J., Hernández J.M., Hofmann W.-K., Mills K.I., Gilkes A., Chiaretti S., Shurtleff S.A., Kipps T.J., Rassenti L.Z., Yeoh A.E., Papenhausen P.R., Liu W.-M., Williams P.M., Foà R. (2010). Clinical utility of microarray-based gene expression profiling in the diagnosis and subclassification of leukemia: report from the International Microarray Innovations in Leukemia Study Group. J. Clin. Oncol..

[47] Tomiatti V., Istvánffy R., Pietschmann E., Kratzat S., Hoellein A., Quintanilla-Fend L., von Bubnoff N., Peschel C., Oostendorp R.A.J., Keller U. (2015). Cks1 is a critical regulator of hematopoietic stem cell quiescence and cycling, operating upstream of Cdk inhibitors. Oncogene.

[48] Kuntimaddi A., Achille N.J., Thorpe J., Lokken A.A., Singh R., Hemenway C.S., Adli M., Zeleznik-Le N.J., Bushweller J.H. (2015). Degree of recruitment of DOT1L to MLL-AF9 defines level of H3K79 di- and tri-methylation on target genes and transformation potential. Cell Rep..

[49] Yeung J., Esposito M.T., Gandillet A., Zeisig B.B., Griessinger E., Bonnet D., So C.W.E. (2010). β-Catenin mediates the establishment and drug resistance of MLL leukemic stem cells. Cancer Cell.

[50] Wang Y., Krivtsov A.V., Sinha A.U., North T.E., Goessling W., Feng Z., Zon L.I., Armstrong S.A. (2010). The Wnt/beta-catenin pathway is required for the development of leukemia stem cells in AML. Science.

[51] Lane S.W., Wang Y.J., Lo Celso C., Ragu C., Bullinger L., Sykes S.M., Ferraro F., Shterental S., Lin C.P., Gilliland D.G., Scadden D.T., Armstrong S.A., Williams D.A. (2011). Differential niche and Wnt requirements during acute myeloid leukemia progression. Blood.

[52] Wang Z., Smith K.S., Murphy M., Piloto O., Somervaille T.C.P., Cleary M.L. (2008). Glycogen synthase kinase 3 in MLL leukaemia maintenance and targeted therapy. Nature.

[53] Nygren M.K., Døsen G., Hystad M.E., Stubberud H., Funderud S., Rian E. (2007). Wnt3A activates canonical Wnt signalling in acute lymphoblastic leukaemia (ALL) cells and inhibits the proliferation of B-ALL cell lines. Br. J. Haematol..

[54] Kim J., Chang W., Jung Y., Song K., Lee I. (2012). Wnt5a activates THP-1 monocytic cells via a β-catenin-independent pathway involving JNK and NF-κB activation. Cytokine.

[55] Milhollen M.A., Traore T., Adams-Duffy J., Thomas M.P., Berger A.J., Dang L., Dick L.R., Garnsey J.J., Koenig E., Langston S.P., Manfredi M., Narayanan U., Rolfe M., Staudt L.M., Soucy T.A., Yu J., Zhang J., Bolen J.B., Smith P.G. (2010). MLN4924, a NEDD8-activating enzyme inhibitor, is active in diffuse large B-cell lymphoma models: rationale for treatment of NF-{kappa}B-dependent lymphoma. Blood.

[56] Soucy T.A., Smith P.G., Rolfe M. (2009). Targeting NEDD8-activated cullin-RING ligases for the treatment of cancer. Clin. Cancer Res..

[57] Wu L., Grigoryan A.V., Li Y., Hao B., Pagano M., Cardozo T.J. (2012). Specific small molecule inhibitors of Skp2-mediated p27 degradation. Chem. Biol..

[58] Pavlides S.C., Huang K.-T., Reid D.A., Wu L., Blank S.V., Mittal K., Guo L., Rothenberg E., Rueda B., Cardozo T., Gold L.I. (2013). Inhibitors of SCF-Skp2/Cks1 E3 ligase block estrogen-induced growth stimulation and degradation of nuclear p27kip1: therapeutic potential for endometrial cancer. Endocrinology.

[59] Yu W., Chory E.J., Wernimont A.K., Tempel W., Scopton A., Federation A., Marineau J.J., Qi J., Barsyte-Lovejoy D., Yi J., Marcellus R., Iacob R.E., Engen J.R., Griffin C., Aman A., Wienholds E., Li F., Pineda J., Estiu G., Shatseva T., Hajian T., Al-Awar R., Dick J.E., Vedadi M., Brown P.J., Arrowsmith C.H., Bradner J.E., Schapira M. (2012). Catalytic site remodelling of the DOT1L methyltransferase by selective inhibitors. Nat. Commun..

[60] Swords R.T., Erba H.P., Deangelo D.J., Bixby D.L., Altman J.K., Maris M., Hua Z., Blakemore S.J., Faessel H., Sedarati F., Dezube B.J., Giles F.J., Medeiros B.C. (2015). Pevonedistat (MLN4924), a first-in-class NEDD8-activating enzyme inhibitor, in patients with acute myeloid leukaemia and myelodysplastic syndromes: a phase 1 study. Br. J. Haematol..

[61] Bailly A., Perrin A., Bou Malhab L.J., Pion E., Larance M., Nagala M., Smith P., O'Donohue M.-F., Gleizes P.-E., Zomerdijk J., Lamond A.I., Xirodimas D.P. (2015). Oncogene.

[62] Hoellein A., Graf S., Bassermann F., Schoeffmann S., Platz U., Hölzlwimmer G., Kröger M., Peschel C., Oostendorp R., Quintanilla-Fend L., Keller U., Holzlwimmer G., Kroger M., Peschel C., Oostendorp R., Quintanilla-Fend L., Keller U., Hölzlwimmer G., Kröger M., Peschel C., Oostendorp R., Quintanilla-Fend L., Keller U. (2012). Cks1 promotion of S phase entry and proliferation is independent of p27Kip1 suppression. Mol. Cell. Biol..

[63] Ng R.K., Kong C.T., So C.C., Lui W.C., Chan Y.F., Leung K.C., So K.C., Tsang H.M., Chan L.C., Sham M.H. (2014). Epigenetic dysregulation of leukaemic HOX code in MLL-rearranged leukaemia mouse model. J. Pathol..

[64] Krivtsov A.V., Armstrong S.A. (2007). MLL translocations, histone modifications and leukaemia stem-cell development. Nat. Rev. Cancer.

[65] Xia Z.B., Popovic R., Chen J., Theisler C., Stuart T., Santillan D.A., Erfurth F., Diaz M.O., Zeleznik-Le N.J. (2005). The MLL fusion gene, MLL-AF4, regulates cyclin-dependent kinase inhibitor CDKN1B (p27kip1) expression. Proc. Natl. Acad. Sci. U. S. A..

[66] Chen W., Kumar A.R., Hudson W.A., Li Q., Wu B., Staggs R.A., Lund E.A., Sam T.N., Kersey J.H. (2008). Malignant transformation initiated by Mll-AF9: gene dosage and critical target cells. Cancer Cell.

[67] Chen C.-W., Koche R.P., Sinha A.U., Deshpande A.J., Zhu N., Eng R., Doench J.G., Xu H., Chu S.H., Qi J., Wang X., Delaney C., Bernt K.M., Root D.E., Hahn W.C., Bradner J.E., Armstrong S.A. (2015). DOT1L inhibits SIRT1-mediated epigenetic silencing to maintain leukemic gene expression in MLL-rearranged leukemia. Nat. Med..

[68] Thiel A.T., Blessington P., Zou T., Feather D., Wu X., Yan J., Zhang H., Liu Z., Ernst P., Koretzky G.A., Hua X. (2010). MLL-AF9-induced leukemogenesis requires coexpression of the wild-type Mll allele. Cancer Cell.

[69] Wong S.H.K.H.K., Goode D.L.L., Iwasaki M., Wei M.C.C., Kuo H.-P.P., Zhu L., Schneidawind D., Duque-Afonso J., Weng Z., Cleary M.L.L. (2015). The H3K4-methyl epigenome regulates leukemia stem cell oncogenic potential. Cancer Cell.

[70] Rathert P., Roth M., Neumann T., Muerdter F., Roe J.-S.J.-S., Muhar M., Deswal S., Cerny-Reiterer S., Peter B., Jude J., Hoffmann T., Boryń Ł.M., Axelsson E., Schweifer N., Tontsch-Grunt U., Dow L.E.L.E.L.E., Gianni D., Pearson M., Valent P., Stark A., Kraut N., Vakoc C.R., Zuber J., Boryn L.M., Axelsson E., Schweifer N., Tontsch-Grunt U., Dow L.E.L.E.L.E., Gianni D., Pearson M., Valent P., Stark A., Kraut N., Vakoc C.R., Zuber J., Boryń Ł.M., Axelsson E., Schweifer N., Tontsch-Grunt U., Dow L.E.L.E.L.E., Gianni D., Pearson M., Valent P., Stark A., Kraut N., Vakoc C.R., Zuber J. (2015). Transcriptional plasticity promotes primary and acquired resistance to BET inhibition. Nature.

[71] Fong C.Y., Gilan O., Lam E.Y.N., Rubin A.F., Ftouni S., Tyler D., Stanley K., Sinha D., Yeh P., Morison J., Giotopoulos G., Lugo D., Jeffrey P., Lee S.C.W., Carpenter C., Gregory R., Ramsay R.G., Lane S.W., Abdel-Wahab O., Kouzarides T., Johnstone R.W., Dawson S.J., Huntly B.J.P., Prinjha R.K., Papenfuss A.T., Dawson M.A. (2015). BET inhibitor resistance emerges from leukaemia stem cells. Nature.

[72] Müller-Tidow C., Steffen B., Cauvet T., Tickenbrock L., Ji P., Diederichs S., Sargin B., Köhler G., Stelljes M., Puccetti E., Ruthardt M., de Vos S., Hiebert S.W., Koeffler H.P., Berdel W.E., Serve H. (2004). Translocation products in acute myeloid leukemia activate the Wnt signaling pathway in hematopoietic cells. Mol. Cell. Biol..

[73] Mikesch J.-H., Steffen B., Berdel W.E., Serve H., Müller-Tidow C. (2007). The emerging role of Wnt signaling in the pathogenesis of acute myeloid leukemia. Leukemia.

[74] Wang J., Han F., Lee S.-W.W., Wu J., Chan C.-H.H., Zhang X., Gao Y., Su H.-K.K., Feng Z.-Z.Z., Xu D.-Z.Z., Lin H.-K.K. (2014). E3-ligase Skp2 regulates β-catenin expression and maintains hematopoietic stem cell homing. Biochem. Biophys. Res. Commun..

[75] Fung T.K., Gandillet A., So C.W.E. (2012). Selective treatment of mixed-lineage leukemia leukemic stem cells through targeting glycogen synthase kinase 3 and the canonical Wnt/β-catenin pathway. Curr. Opin. Hematol..

[76] Shah B.D., Zuckerman K.S. (2011). Normal and malignant hematopoiesis. Adv. Malig. Hematol.

[77] Dietrich P.A., Yang C., Leung H.H.L., Lynch J.R., Gonzales E., Liu B., Haber M., Norris M.D., Wang J.Y.J.Y., Wang J.Y.J.Y. (2014). GPR84 sustains aberrant-catenin signaling in leukemic stem cells for maintenance of MLL leukemogenesis. Blood.

[78] Hulit J., Lee R.J., Li Z., Wang C., Katiyar S., Yang J., Quong A.A., Wu K., Albanese C., Russell R., Di Vizio D., Koff A., Thummala S., Zhang H., Harrell J., Sun H., Muller W.J., Inghirami G., Lisanti M.P., Pestell R.G. (2006). p27Kip1 repression of ErbB2-induced mammary tumor growth in transgenic mice involves Skp2 and Wnt/beta-catenin signaling. Cancer Res..

[79] Philipp-Staheli J., Payne S.R., Kemp C.J. (2001). p27(Kip1): regulation and function of a haploinsufficient tumor suppressor and its misregulation in cancer. Exp. Cell Res..

[80] Glover C.E., Gurley K.E., Kim K.-H., Storer B., Fero M.L., Kemp C.J. (2009). Endocrine dysfunction in p27Kip1 deficient mice and susceptibility to Wnt-1 driven breast cancer. Carcinogenesis.

[81] Qi J., Yu Y., Akilli Öztürk Ö., Holland J.D., Besser D., Fritzmann J., Wulf-Goldenberg A., Eckert K., Fichtner I., Birchmeier W. (2015). New Wnt/β-catenin target genes promote experimental metastasis and migration of colorectal cancer cells through different signals. Gut.

[82] Flotho C., Coustan-smith E., Pei D., Cheng C., Song G., Pui C.-H., Downing J.R.J., Campana D., Pui C.-H., Evans W., Riehm H., Reiter A., Schrappe M., Lilleyman J., Gibson B., Stevens R., Steinherz P., Gaynon P., Breneman J., Sandlund J., Harrison P., Rivera G., Szczepanski T., Orfao A., van der Velden V., Miguel J.S., van Dongen J., Campana D., Coustan-smith E., Behm F., Sanchez J., van Dongen J., Seriu T., Panzer-Grumayer E., Coustan-smith E., Sancho J., Hancock M., Biondi A., Valsecchi M., Seriu T., Golub T., Slonim D., Tamayo P., Yeoh E., Ross M., Shurtleff S., Armstrong S., Staunton J., Silverman L., Ross M., Zhou X., Song G., Haferlach T., Kohlmann A., Schnittger S., Andersson A., Olofsson T., Lindgren D., Holleman A., Cheok M., den Boer M., Lugthart S., Cheok M., den Boer M., Pui C.-H., Sandlund J., Pei D., Pui C.-H., Pei D., Sandlund J., Pui C.-H., Relling M., Sandlund J., Downing J.R.J., Campana D., Evans W., Flotho C., Coustan-smith E., Pei D., Coustan-smith E., Sancho J., Behm F., Cheng C., Pounds S., Boyett J., Fine J., Gray J., Greaves M., Panzer-Grumayer E., Schneider M., Panzer S., Fasching K., Gadner H., Towatari M., Adachi K., Marunouchi T., Saito H., MacGrogan G., Rudolph P., Mascarel I., Durbecq V., Paesmans M., Cardoso F., Guerin E., Entz-Werle N., Eyer D., Klumper E., Giaccone G., Pieters R., Wang X., Jin D., Wong H., Swanton C., Tomlinson I., Downward J., Cario G., Stanulla M., Fine B., Bjorck E., Ek S., Landgren O., Laub F., Aldabe R., Friedrich V., Frei E., Sallan S., Brisco M., Sykes P., Dolman G., Bhojwani D., Kang H., Moskowitz N. (2007). A set of genes that regulate cell proliferation predicts treatment outcome in childhood acute lymphoblastic leukemia. Blood.

[83] Andersson A.K., Ma J., Wang J., Chen X., Gedman A.L., Dang J., Nakitandwe J., Holmfeldt L., Parker M., Easton J., Huether R., Kriwacki R., Rusch M., Wu G., Li Y., Mulder H., Raimondi S., Pounds S., Kang G., Shi L., Becksfort J., Gupta P., Payne-Turner D., Vadodaria B., Boggs K., Yergeau D., Manne J., Song G., Edmonson M., Nagahawatte P., Wei L., Cheng C., Pei D., Sutton R., Venn N.C., Chetcuti A., Rush A., Catchpoole D., Heldrup J., Fioretos T., Lu C., Ding L., Pui C.-H., Shurtleff S., Mullighan C.G., Mardis E.R., Wilson R.K., Gruber T. a, Zhang J., Downing J.R. (2015). The landscape of somatic mutations in infant MLL-rearranged acute lymphoblastic leukemias. Nat. Genet..

[84] Greaves M. (2015). When one mutation is all it takes. Cancer Cell.

[85] Yokoyama A. (2015). Molecular mechanisms of MLL-associated leukemia. Int. J. Hematol..

[86] Meyer C., Hofmann J., Burmeister T., Gröger D., Park T.S., Emerenciano M., Pombo de Oliveira M., Renneville A., Villarese P., Macintyre E., Cavé H., Clappier E., Mass-Malo K., Zuna J., Trka J., De Braekeleer E., De Braekeleer M., Oh S.H., Tsaur G., Fechina L., van der Velden V.H.J., van Dongen J.J.M., Delabesse E., Binato R., Silva M.L.M., Kustanovich A., Aleinikova O., Harris M.H., Lund-Aho T., Juvonen V., Heidenreich O., Vormoor J., Choi W.W.L., Jarosova M., Kolenova A., Bueno C., Menendez P., Wehner S., Eckert C., Talmant P., Tondeur S., Lippert E., Launay E., Henry C., Ballerini P., Lapillone H., Callanan M.B., Cayuela J.M., Herbaux C., Cazzaniga G., Kakadiya P.M., Bohlander S., Ahlmann M., Choi J.R., Gameiro P., Lee D.S., Krauter J., Cornillet-Lefebvre P., Te Kronnie G., Schäfer B.W., Kubetzko S., Alonso C.N., Zur Stadt U., Sutton R., Venn N.C., Izraeli S., Trakhtenbrot L., Madsen H.O., Archer P., Hancock J., Cerveira N., Teixeira M.R., Lo Nigro L., Möricke A., Stanulla M., Schrappe M., Sedék L., Szczepański T., Zwaan C.M., Coenen E.A., van den Heuvel-Eibrink M.M., Strehl S., Dworzak M., Panzer-Grümayer R., Dingermann T., Klingebiel T., Marschalek R. (2013). The MLL recombinome of acute leukemias in 2013. Leukemia.

[87] Soucy T.A., Smith P.G., Milhollen M.A., Berger A.J., Gavin J.M., Adhikari S., Brownell J.E., Burke K.E., Cardin D.P., Critchley S., Cullis C.A., Doucette A., Garnsey J.J., Gaulin J.L., Gershman R.E., Lublinsky A.R., McDonald A., Mizutani H., Narayanan U., Olhava E.J., Peluso S., Rezaei M., Sintchak M.D., Talreja T., Thomas M.P., Traore T., Vyskocil S., Weatherhead G.S., Yu J., Zhang J., Dick L.R., Claiborne C.F., Rolfe M., Bolen J.B., Langston S.P. (2009). An inhibitor of NEDD8-activating enzyme as a new approach to treat cancer. Nature.

[88] Milhollen M.A., Thomas M.P., Narayanan U., Traore T., Riceberg J., Amidon B.S., Bence N.F., Bolen J.B., Brownell J., Dick L.R., Loke H.-K., McDonald A.A., Ma J., Manfredi M.G., Sells T.B., Sintchak M.D., Yang X., Xu Q., Koenig E.M., Gavin J.M., Smith P.G. (2012). Treatment-emergent mutations in NAEβ confer resistance to the NEDD8-activating enzyme inhibitor MLN4924. Cancer Cell.

[89] Ganoth D., Bornstein G., Ko T.K., Larsen B., Tyers M., Pagano M., Hershko A. (2001). The cell-cycle regulatory protein Cks1 is required for SCF(Skp2)-mediated ubiquitinylation of p27. Nat. Cell Biol..

[90] Calvisi D.F., Ladu S., Pinna F., Frau M., Tomasi M.L., Sini M., Simile M.M., Bonelli P., Muroni M.R., Seddaiu M.A., Lim D.S., Feo F., Pascale R.M. (2009). SKP2 and CKS1 promote degradation of cell cycle regulators and are associated with hepatocellular carcinoma prognosis. Gastroenterology.

[91] Khattar V., Thottassery J.V. (2013). Cks1: structure, emerging roles and implications in multiple cancers. J. Cancer Ther..

[92] Zhao H., Lu Z., Bauzon F., Fu H., Cui J., Locker J., Zhu L. (2017). p27T187A knockin identifies Skp2/Cks1 pocket inhibitors for advanced prostate cancer. Oncogene.

[93] Dawson M.A., Prinjha R.K., Dittmann A., Giotopoulos G., Bantscheff M., Chan W.-I., Robson S.C., Chung C., Hopf C., Savitski M.M., Huthmacher C., Gudgin E., Lugo D., Beinke S., Chapman T.D., Roberts E.J., Soden P.E., Auger K.R., Mirguet O., Doehner K., Delwel R., Burnett A.K., Jeffrey P., Drewes G., Lee K., Huntly B.J.P., Kouzarides T. (2011). Inhibition of BET recruitment to chromatin as an effective treatment for MLL-fusion leukaemia. Nature.

[94] Daigle S.R., Olhava E.J., Therkelsen C.A., Majer C.R., Sneeringer C.J., Song J., Johnston L.D., Scott M.P., Smith J.J., Xiao Y., Jin L., Kuntz K.W., Chesworth R., Moyer M.P., Bernt K.M., Tseng J.-C., Kung A.L., Armstrong S.A., Copeland R.A., Richon V.M., Pollock R.M. (2011). Selective killing of mixed lineage leukemia cells by a potent small-molecule DOT1L inhibitor. Cancer Cell.

[95] Daigle S.R., Campbell C.T., Waters N.J., Olhava E.J., Copeland R.A., Blakemore S.J., Pollock R.M., Smith J.J. (2015). Abstract 2701: characterization of acquired EPZ-5676 resistance in cell line models of MLL rearranged leukemia. Cancer Res..

[96] Haladyna J., Yamauchi T., Neff T., Bernt K.M. (2014). 3622 MDR1 Mediated Drug Resistance to a Histone Methyltransferase Inhibitor (KMT), in: 56th ASH Annu. Meet. https://ash.confex.com/ash/2014/webprogram/Paper75364.html.

[97] Hamdi A., Lesnard A., Suzanne P., Robert T., Miteva M. a, Pellerano M., Didier B., Ficko-Blean E., Lobstein A., Hibert M., Rault S., Morris M.C., Colas P. (2015). Tampering with cell division by using small-molecule inhibitors of CDK-CKS protein interactions. Chembiochem.

[98] Lv M., Zhang X., Li M., Chen Q., Ye M., Liang W., Ding L., Cai H., Fu D., Lv Z., Gonzalez-Gonzalez R., Bologna-Molina R., Carreon-Burciaga R., Gomezpalacio-Gastelum M., Molina-Frechero N., Bartel D., Chen C., Schmittgen T., Livak K., Pallante P., Visone R., Croce C., Fusco A., He H., Jazdzewski K., Li W., Liyanarachchi S., Nagy R., Tetzlaff M., Liu A., Xu X., Master S., Baldwin D., Mitomo S., Maesawa C., Ogasawara S., Iwaya T., Shibazaki M., Visone R., Pallante P., Vecchione A., Cirombella R., Ferracin M., Chen H., Chen G., Chen Y., Liao W., Liu C., Dang X., Ma A., Yang L., Hu H., Zhu B., Huse J., Brennan C., Hambardzumyan D., Wee B., Pena J., Lan Y., Zhang Y., Wang J., Lin C., Ittmann M., Martinsson-Ahlzen H., Liberal V., Grunenfelder B., Chaves S., Spruck C., Ban Y., Yamamoto G., Takada M., Hayashi S., Shimizu K., Lu J., He M., Wang L., Chen Y., Liu X., Ji J., Shi J., Budhu A., Yu Z., Forgues M., Sander S., Bullinger L., Klapproth K., Fiedler K., Kestler H., Hayles J., Beach D., Durkacz B., Nurse P., Richardson H., Stueland C., Thomas J., Russell P., Reed S., Doree M., Hunt T., Vassilev L., Tovar C., Chen S., Knezevic D., Zhao X. (2013). miR-26a and its target CKS2 modulate cell growth and tumorigenesis of papillary thyroid carcinoma. PLoS One.

[99] Hu M., Wang M., Lu H., Wang X., Fang X., Wang J., Ma C., Chen X., Xia H., Hu M., Wang M., Lu H., Wang X., Fang X., Wang J., Ma C., Chen X., Xia H. (2016). Loss of miR-1258 contributes to carcinogenesis and progression of liver cancer through targeting CDC28 protein kinase regulatory subunit 1B. Oncotarget.

