# Supplementary material for: The Cks1/Cks2 axis fine-tunes Mll1 expression and is crucial for MLL-rearranged leukaemia cell viability
Source: Biochim Biophys Acta. 2018 Jan;1865(1):105–16. doi: 10.1016/j.bbamcr.2017.09.009 (PMC5701546; doi:10.1016/j.bbamcr.2017.09.009)
Supplement: Supplementary file 2 — Supplementary material [file mmc1.docx]

**SUPPLEMENTAL INFORMATION FOR:**

**The Cks1/Cks2 axis fine-tunes Mll1 expression and is crucial for MLL-rearranged leukaemia cell viability**

William Grey^1,2^, Adam Ivey^1,3^, Thomas A. Milne^4^, Torsten Haferlach^5^, David Grimwade^1§^, Frank Uhlmann^6^, Edwige Voisset^1*^ & Veronica Yu^1*§^

**Supplementary Table S1**

**Supplementary Figure S1**

**Figure S1.** *Cks*-deficient spontaneously immortalised MEFs confirm previously published phenotypes [8]. (A) Cell cycle analysis of *Cks*-deficient MEFs was determined using the Click-iT EdU assay. (B) Western blots for whole cell lysates from *Cks*-deficient MEFs. Bands were quantified by ImageJ. (C) Confocal microscopy for MEFs confirming γH2AX phenotype (DAPI-blue, γH2AX-green). Images were taken using a Zeiss Axioplan-II microscope at x63 magnification. All panels are representative of 3 independent experiments.

**Supplementary Figure S2**

**Figure S2.** Confirmation of *Mll1* knockdown efficiency by siRNA in MEFs. (A) mRNA level for *Mll1* knockdown. *Actin* and *Gapdh* were used as control genes for qPCR, and a Student’s *t*-test was used to analyse significance of difference. (B) Western blot for Mll1 knockdown. Histone H3 was used as a nuclear loading control. (C) Confirmation of *MLL1* overexpression in MEFs by qPCR. *Actin* and *Gapdh* were used as control genes for qPCR, and a Student’s *t*-test was used to analyse significance of difference.

**Supplementary Figure S3**

**Figure S3.** ImageStream^X^ analysis workflow. (A) Gating strategy isolating single, in focus, and double positive (DAPI/β-catenin) cells. (B) Image mask applied to cells covering the brightfield area for analysis: Object(M01, Brightfield, Tight). Top panel represents gated cells selected for analysis. Bottom panel includes the object mask superimposed in cyan. Scale bar, 10μm.

**Supplementary Figure S4**

**Figure S4.** ImageStream^X^ representative images for β-catenin nuclear translocation. WT and *Cks*-deficient MEFs were treated with 50ng/mL Wnt3a for the indicated time points, and with Non-Specific- or *Mll1*-siRNA (11 and 14). Images are representative of 3 independent experiments. Scale bar,10μm.

**Supplementary Figure S5**

**Figure S5.** MLL-FPs heterogeneously retain CKS interactions. *MLL*-translocation carrying cell lines (A) THP-1 (*MLL-AF9*), (B) ML-2 (*MLL-AF6*) and (C) KOPN-8 (*MLL-ENL*) were lysed in RIPA buffer, immunoprecipitated for the endogenous *MLL-*translocation partner, and western blotted for MLL-FPs, CKS1 and CKS2. Isotype controls (IgG) for each antibody were used to confirm specificity of interactions. Western blots are representative of 3 independent experiments.

**Supplementary Figure S6**

**Figure S6.** Comparison of Wnt signalling activity in THP-1 and KOPN-8 cells using the TOPFlash TCF/LEF reporter construct. (A) Relative luciferase activity (RLA) of KOPN-8 and THP-1 cells treated with the indicated siRNA for comparison. (B) Fold change RLA compared to Non-Specific siRNA in KOPN-8 cells showing comparison of two independent siRNAs for *CKS1B* and *CKS2*. (C) Fold change RLA compared to Non-Specific siRNA in THP-1 cells showing comparison of two independent siRNAs for *CKS1B* and *CKS2*. A Student’s *t*-test was used to analyse significance of difference (N=3). * indicates p<0.05, ** indicates p<0.005, and *** indicates p<0.0005.

**Supplementary Figure S7**

**Figure S7.** Knockdown of *CKS1B* and *CKS2* in *MLL*-translocation cell lines. CKS1B mRNA (A) and protein (B) levels, and CKS2 mRNA (C) and protein (D) levels were assayed in THP-1 and KOPN-8 cell lines in response to two independent siRNAs for each gene. *ABL, GUS* and *B2M* were used as control genes for qPCR, Actin was used as a control for western blots, and a Student’s *t*-test was used to analyse significance of difference. * indicates p<0.05, ** indicates p<0.005, and *** indicates p<0.0005. Western blot bands were quantified by ImageJ.

**Supplementary Figure S8**

**Figure S8.** C1 or MLN4924 treatment reduces clonogenicity of human *MLLr* AML, but not CD34^+^ cells. Colony formation assay of CD34^+^, THP-1 and KOPN-8 cells. Student’s *t*-test was used to analyse significance of difference (N=3). * indicates significant differences versus DMSO vehicle control in THP-1 cells. § indicates significant differences versus DMSO vehicle control in KOPN-8.

**Supplementary Figure S9**

**Figure S9.** Assessment of Caspase activation. Western blots for cleaved-Caspase 3 in THP-1 and KOPN-8 cell lines in response to MLN4924, C1 or DMSO vehicle control. Tubulin was used as a loading control.

**Supplementary Figure S10**

**Figure S10.** (A) Cell viability was assessed for ML-1 (MLL-AF6) and RS4;11 (MLL-AF4) cell lines after 3 days treatment with indicated concentrations of MLN4924 or C1. (B) Proportion of apoptotic ML-1 and RS4;11 cells, in response to C1 or MLN4924, was measured by PI and Annexin V-FITC staining using flow cytometry. Significance was tested using the Student’s *t*-test versus DMSO treated cells.
